# CENPA acts as a prognostic factor that relates to immune infiltrates in gliomas

**DOI:** 10.3389/fneur.2022.1015221

**Published:** 2022-10-19

**Authors:** Bo Wang, Wei Wei, Shengrong Long, Lesheng Wang, Bin Yang, Du Wu, Zhengwei Li, Zhiqiang Li, Muhammad Arshad, Xiang Li, Jincao Chen

**Affiliations:** ^1^Department of Neurosurgery, Zhongnan Hospital of Wuhan University, Wuhan, China; ^2^Brain Research Center, Zhongnan Hosptial of Wuhan University, Wuhan, China; ^3^Department of Biological Sciences, International Islamic University Islamabad, Islamabad, Pakistan

**Keywords:** glioma, CENPA, tumor-infiltrating, TCGA, CGGA

## Abstract

**Background:**

Glioma is the most common primary tumor of the central nervous system (CNS). Centromere protein A (CENPA) plays an essential role in ensuring that mitosis proceeds normally. The effect of CENPA on glioma is rarely reported. However, the current study aims to explore whether aberrant CENPA expression promotes glioma progression and the potential mechanisms involved.

**Methods:**

The GEPIA website, The Cancer Genome Atlas, and the Gene Expression Omnibus (GEO) were used to assess the expression of CENPA in glioma. The results were validated by real-time quantitative polymerase chain reaction and immunohistochemical staining of clinical samples. The relationship between the expression and prognostic value of the CENPA gene in glioma was investigated by Kaplan–Meier (KM) survival analysis with RNA-seq and clinical profiles downloaded from the Chinese Glioma Genome Atlas (CGGA) and UCSC Xena. The association between CENPA and clinical characteristics was also evaluated. Cell Counting Kit-8 (CCK8) assay, wound healing assay using two glioma cell lines, gene set enrichment analysis (GSEA), KEGG and gene ontology (GO) enrichment analysis, immune infiltration analysis, temozolomide (TMZ) sensitivity analysis, and single-cell sequence analysis were performed to explore the underlying mechanisms of high CENPA expression and its effect on glioma development. Finally, we performed a Cox analysis based on the expression of CENPA to predict patient prognosis.

**Results:**

CENPA was significantly upregulated in glioma tissue samples and correlated with patient prognosis. Moreover, the downregulation of CENPA inhibited the migration and proliferation of glioma cells. In addition, the expression level of CENPA was significantly correlated with the grade, primary–recurrent–secondary (PRS) type, IDH mutation status, and 1p19q codeletion status. Furthermore, CENPA could serve as an independent prognostic factor for glioma that mainly interferes with the normal progression of mitosis and regulates the tumor immune microenvironment favoring glioma development.

**Conclusion:**

CENPA may act as a prognostic factor in patients with glioma and provide a novel target for the treatment of gliomas.

## Introduction

Glioma is the most common malignant tumor of the CNS, accounting for ~30% of primary CNS malignancies (1). According to the fourth edition of the 2016 WHO Classification of Tumors of the Central Nervous System, IDH mutation and 1p/19q deletion were introduced as new criteria for the classification of gliomas based on the original histologic classification (2, 3). The new molecular-histologic classification is more conducive to treating patients than the original histologic classification. Currently, patients with glioma are primarily treated with a combination of surgery, chemotherapy, and radiotherapy, but the prognosis remains unsatisfactory (4). Thus, discovering novel prognostic markers and therapeutic targets is of great importance for this type of cancer.

CENPA is a histone H3-like variant of centromeric nucleosomes essential for forming centromeres and corresponding kinetochores (5). It plays a key role in cell cycle regulation, cell division, and genetic stability, by ensuring the proper formation and function of the kinetochore during mitosis (6, 7). Overexpression can lead to mislocalization of CENPA on chromosomes and subsequent formation of ectopic neo-centromeres and kinetochores in the chromosome arms. These ectopic structures disrupt the normal segregation of chromosomes during cell division, forming aneuploids and thus leading to tumorigenesis (3, 8). In addition, there is a direct correlation between overexpression of CENPA and genomic instability, which can cause cancer and promote disease progression (9). Previous studies have revealed a significant correlation between CENPA and the survival of patients with glioma (10). In the current study, we extracted information on glioma from TCGA and CGGA databases to investigate whether CENPA can influence glioma progression and the potential mechanisms involved.

## Materials and methods

### Data download and preprocessing

RNA-seq data and corresponding clinical data of patients with glioma that included low-grade glioma (LGG) and glioblastoma (GBM) (mRNAseq_693 dataset and mRNAseq_325 dataset) were downloaded from the CGGA (http://www.cgga.org.cn/). limma (11) and sva (12) packages in R software (R version 4.1.0: https://www.rproject.org/) were used to correct the two sets of gene expression data in batches and integrate them. Otherwise, TCGA TARGET GTEx cohort from UCSC Xena (https://ucsc.xena.edu) was downloaded to extract gene expression data and corresponding clinical data of 523 patients with LGG and 171 GBM. In addition, gene expression data of 105 normal brain cortex tissues were extracted to be used as controls. A total of two glioma datasets GSE4290 and GSE16011 from the Gene Expression Omnibus database (http://www.ncbi.nlm.nih.gov/geo/) were downloaded to verify whether CENPA was differentially expressed in normal tissues and gliomas.

### Human glioma and normal brain tissues

Gliomas and normal brain tissues were obtained from patients undergoing surgical resections at Zhongnan Hospital of Wuhan University after obtaining informed consents from them. Gliomas were diagnosed based on the 2016 WHO Classification of Tumors of the Central Nervous System. The use of these glioma and normal samples was approved by the Ethics Committee of the Zhongnan Hospital of Wuhan University (no. 2019048).

### RNA extraction, CDNA synthesis, and quantitative real-time PCR

Takara RNAiso Plus (Takara Bio. Inc., Otsu, Shiga, Japan) was used to isolate total RNA from the tissues persevered at a temperature <−80°C according to the manufacturer's protocol. cDNA was synthesized from 250 ng RNA according to the HiScriptIIQ RT SuperMix for qPCR Kit (Vazyme Medical Technology). The 2^−Δ*Ct*^ method was used to process qPCR data. Primer sequences used were listed as follows: GAPDH-F, 5′-GGAGCGAGATCCCTCCAAAAT-3′, GAPDH-R,5′-GGCTGTTGTCATACTTCTCATGG-3′; CENPA-F,5'CTTAGGCGCTTCCTCCCATC-3′, CENPA-R, 5′- AATGCTTCTGCTGCCAGG-3′.

### Immunohistochemistry

Human glioma tissues fixed in neutral buffered formalin (G1101, Servicebio, Wuhan, China) for 24 h were processed for paraffin embedding. After that, the glioma tissues were cut into 5-μm sections; the glioma sections were then deparaffinized, rehydrated, and immersed successively. Blocking was performed by incubating the sections in NCM Blot Blocking Buffer (P30500, NCM Biotech, Suzhou, China) for 1 h. After washing three times with PBS, the glioma sections were incubated overnight with a specific primary antibody (A15995, ABclonal, Wuhan, China) against CENPA at 4°C. Then, the sections were washed and incubated with poly-HRP-conjugated Goat Anti-Rabbit IgG (H+L) (RCA054, Recordbio Biological Technology, Shanghai, China) at room temperature for 2 h. After being stained with fresh 3, 3′-diaminobenzidine solution and hematoxylin counterstain at room temperature, the slides were observed by light microscopy. The percentage of positive cells was scored for analysis.

### Cell culture and transfection

The glioma cell lines including U251 and T98G purchased from the Cell Library of the Chinese Academy of Sciences (Shanghai, China) were cultured in Dulbecco's modified Eagle medium (DMEM; Servicebio) with 10% fetal bovine serum (FBS, LONSERA) and 10 ul/ml penicillin–streptomycin (Biosharp) at a humidified chamber at 37°C with 5% CO_2_. The sense sequence of small interfering (si) RNA against human CENPA (5′-GGGAUUCGGGUUCGUAACU-3′), a non-targeting control siRNA, and RNA TransMate (E607402) were obtained from Sangon Biotech (Shanghai, China). The transfection procedure was according to the manufacturer's protocol. After 24 h of transfection, the cells were used for further experiments.

### Cell counting Kit-8 proliferation assay

CCK8 assay was used to evaluate cell proliferation according to the manufacturer's protocol (A311-01, Vazyme Biotech, Nanjing, China). U251 and T98G cells were seeded in 96-well-plates at a density of 5,000 cells/100 ul/well. Then, 10 ul of CCK8 solution was added to each well of the plates and incubated for 1 h at 37°C. Finally, the absorbance was analyzed at 450 nM by using a BioTek Synergy HT Microplate Reader.

### Wound healing assay

Wound healing assay was performed to evaluate cell migration activity. U251 and T98G cells were seeded in 12-well-plates at a density of 500,000 cells/1 ml/well. After 24 h, a 10-ul disposable pipette tip was run over the surface of the cells to create scratches. The cells were washed three times with PBS and cultured with 3% FBS concentration medium. The extent of wound healing was measured at 0 and 24 h, respectively.

### Bioinformatics analysis

The survival (https://CRAN.R-project.org/package=survival) and survminer packages (https://cran.r-project.org/web/packages/survivalminer) in R software were used to draw the Kaplan–Meier curve using data of patients with glioma obtained from the CGGA database. Meanwhile, univariate Cox analysis and multivariate Cox analysis were performed. The survival ROC package (https://CRAN.R-project.org/package=survivalROC) was also used to plot a receiver operating characteristic curve (ROC) survival curve using the data of patients with glioma retrieved from the CGGA and TCGA databases, separately. The clinical characteristics obtained from the CGGA were assessed by R to extract those significantly related to CENPA expression. The clusterProfiler package (13) was used to perform KEGG and GO enrichment analysis.

### Gene set enrichment analysis

GSEA, a gene set-based enrichment analysis method, was performed for enrichment analysis of gene function based on the correlation of gene expression data with the phenotype (14). GESA 4.1.0 was downloaded from the Broad Institute website (http://software.broadinstitute.org/gsea/index.jsp), and the pathways to be analyzed were obtained from c2.cp.kegg.v7.0.symbols.gmt dataset in Molecular Signature Database (MsigDB). The weighted enrichment method was used for analysis, with the number of random combinations set to 1,000, and all other parameters set to default. Gene sets with a *p* < 0.05 and a false discovery rate (FDR) < 0.05 were considered significantly enriched genes.

### Immune infiltration analysis

CIBERSORT (15) and ESTIMATE (16) packages were used to calculate the proportion of different immune-infiltrating cells and immune scores and stromal scores in patients with glioma.

### Single-cell RNA-seq data analysis

The single-cell RNA-seq dataset GSE182109 was downloaded from the GEO datasets. Some low-quality sequencing results were excluded based on the criterion of <200 expressed genes or 20% mitochondrial transcripts. Finally, 30,860 genes and 250,944 cells were left. Seurat V4.0 (17) and Harmony V1.0 (18) were used to normalize, cluster, and batch-correct single cells.

### Statistical analysis

Statistical analysis was performed in R v.4.1.0 and GraphPad Prism v.9.0 (GraphPad Software, La Jolla, California). The Wilcoxon rank-sum test and Student's *t*-test for continuous variables and Pearson chi-square test for categorical variables were used to compare various parameters in TCGA, CGGA, and GEO datasets. Overall survival was analyzed by using the Kaplan–Meier method. ROC curve analysis was used to predict overall survival with R package “pROC” (19). Univariate and multivariate Cox regression analyses were used to determine independent prognostic factors. A *P* < 0.05 was considered statistically significant.

## Results

### CENPA is highly expressed in glioma and correlates with patients' prognosis

**Figure 1:**
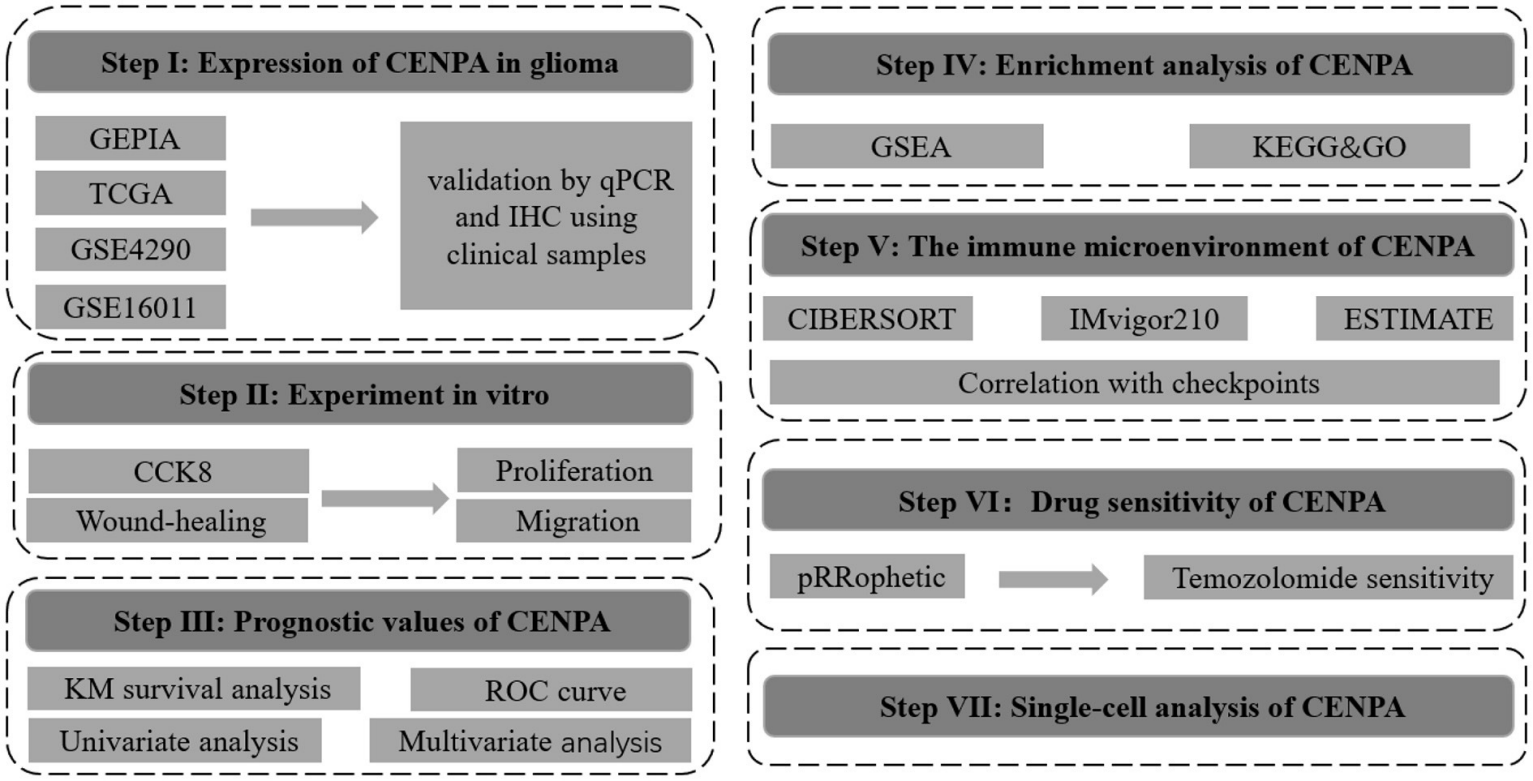
Flowchart of this study.

**Figure 2:**
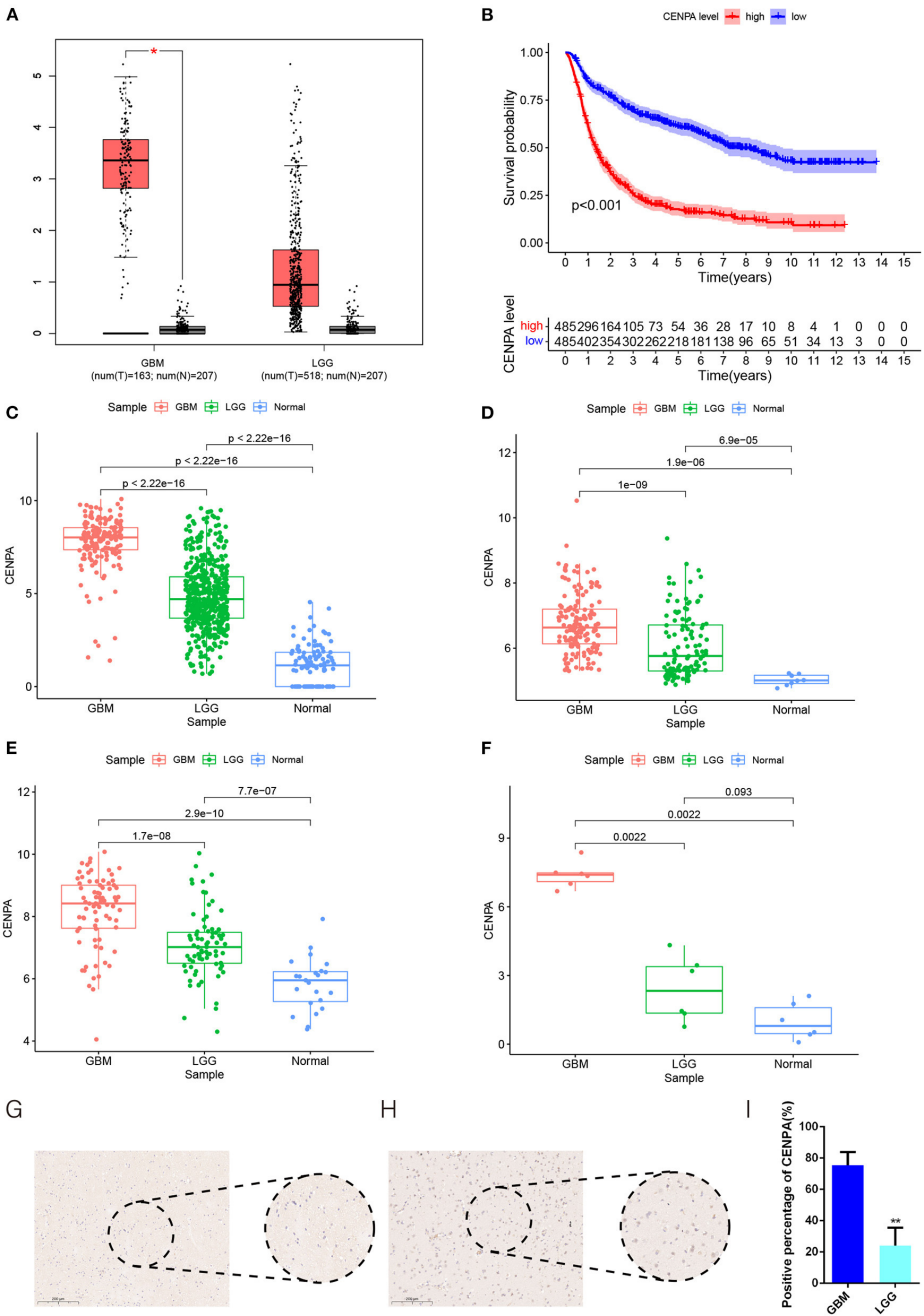
CENPA expression is upregulated in patients with glioma and suggests a poor prognosis. **(A)** Expression of CENPA in glioma and normal tissues in the GEPIA database (one-way ANOVA, *P < 0.05). **(B)** Survival analysis of patients with glioma in the high CENPA and low CENPA groups. Red indicates high expression and blue indicates low expression. ***P < 0.001. The expression of CENPA increased sequentially in normal samples, low-grade gliomas, and high-grade gliomas in the data retrieved from TCGA **(C)**, GSE4290 **(D)**, GSE16011 **(E)**, and samples obtained from our institute **(F)** (Wilcoxon test, *P < 0.05, **P < 0.01, ***P < 0.001). **(G,H)** Immunohistochemistry of CENPA protein levels in low-grade glioma tissues **(G)** and glioblastoma tissues **(H)**. **(I)** Immunohistochemistry revealed higher expression patterns of CENPA in glioblastoma tissues than in low-grade glioma tissues (Student's *t*-test, *P < 0.05, **P < 0.01, ***P < 0.001).

The flow chart of this study was shown in Figure 1. The expression of CENPA in glioma and normal tissue samples was evaluated using the GEPIA website, and the result revealed that CENPA was significantly expressed in both LGG and GBM tissues (Figure 2A). The Kaplan–Meier survival analysis of the CGGA dataset (including GBM and LGG) revealed that the lower the expression of CENPA, the better the prognosis of patients with glioma (Figure 2B). We used TCGA TARGET GTEx cohort downloaded from UCSC Xena to evaluate CENPA expression in the glioma and normal tissue samples. A total of 105 normal brain cortex samples were selected as the control group. The results showed that CENPA expression was sequentially higher in the LGG and GBM tissues than in the normal tissues (Figure 2C). The differential expression of CENPA among the glioma and normal tissues was further validated using the gene expression information extracted from GSE4290 (Figure 2D) and GSE16011 (Figure 2E). Meanwhile, qPCR results of patients with gliomas from our hospital also validated the differential expression of CENPA in normal brain tissues compared with glioma tissues (Figure 2F). Although the difference between low-grade glioma and normal tissues was not significant due to the small sample size, it was still the same trend as in the public database. Immunohistochemical results from another batch of patients' tissues showed that CENPA was also significantly upregulated at the protein level in GBM tissues compared to LGG tissues (Figures 2G–I).

### CENPA inhibition reduced glioma proliferation and migration

**Figure 3:**
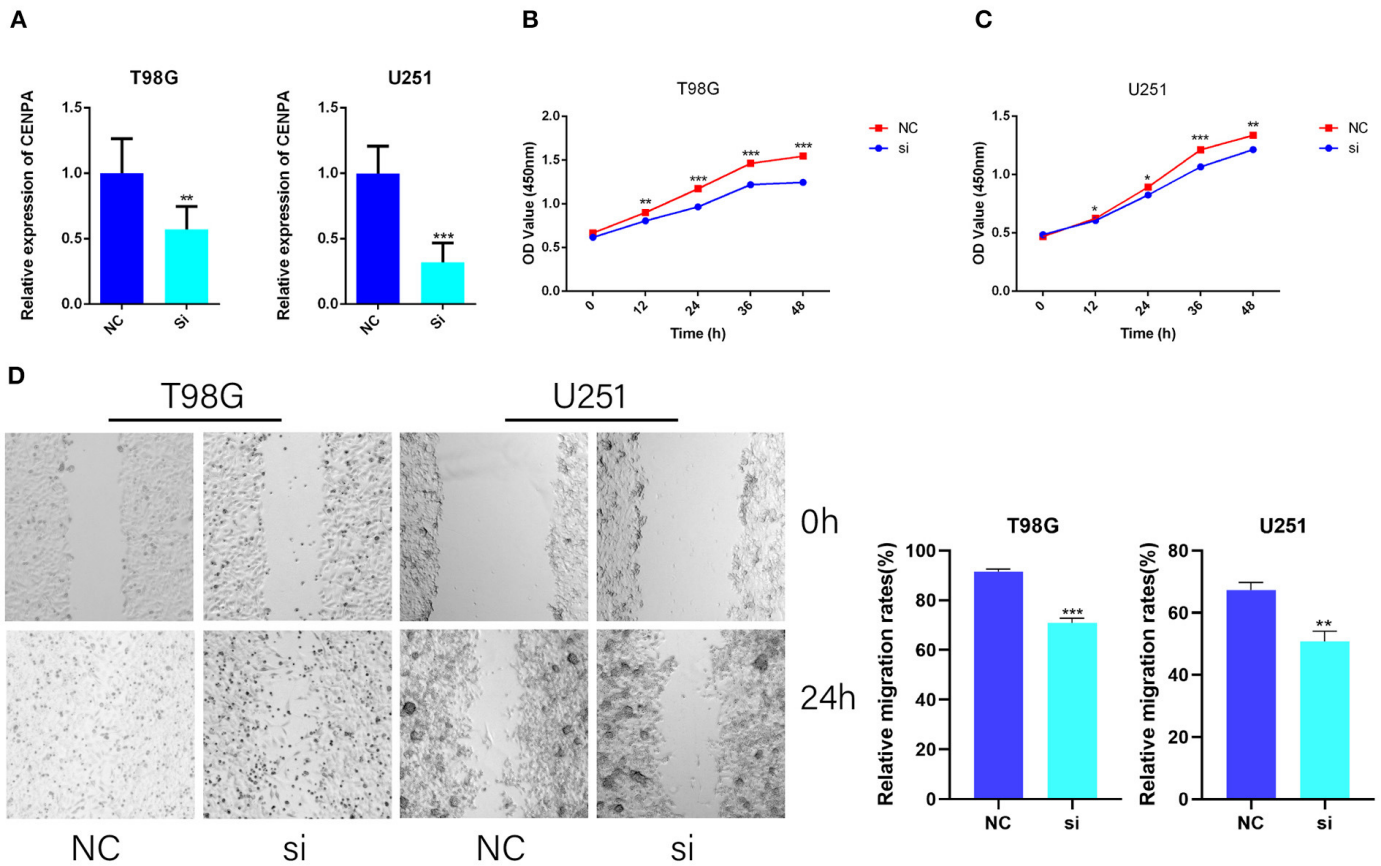
CENPA promotes T98G and U251 cell viability and migration. T98G and U251 were transfected with CENPA-siRNA and NC, respectively. **(A)** siRNA effectively inhibits the expression of CENPA in U251 cells and T98G cells. **(B,C)** Cell viability was measured by the CCK8 assay in T98G and U251, separately (Student's *t*-test, *P < 0.05, **P < 0.01, ***P < 0.001). **(D)** Cell migration was measured by a wound healing assay (Student's *t*-test, *P < 0.05, **P < 0.01, ***P < 0.001). Each experiment was repeated three times with similar results.

To further validate the potential oncogenic role of CENPA in gliomas, CCK8 and wound healing assays were performed in T98G and U251 cell lines. The siRNA sequence against CENPA was designed and assessed for its knockdown efficiency in U251 and T98G cells (Figure 3A). The CCK8 assay was performed to assess the role of CENPA in glioma cell proliferation. The downregulation of CENPA resulted in a significant decrease in OD450 values in both T98G (Figure 3B) and U251 (Figure 3C) cells 48 h after transfection. To assess the function of CENPA in cell migration, the wound healing assay was performed, and the result showed that knockdown of CENPA significantly decreased the wound healing rate in both U251 and T98G cells at 24 h (Figure 3D), suggesting that downregulation of CENPA could inhibit the migration of glioma cells *in vitro*.

### Relationship analysis between CNEPA expression and clinical characteristics

**Figure 4:**
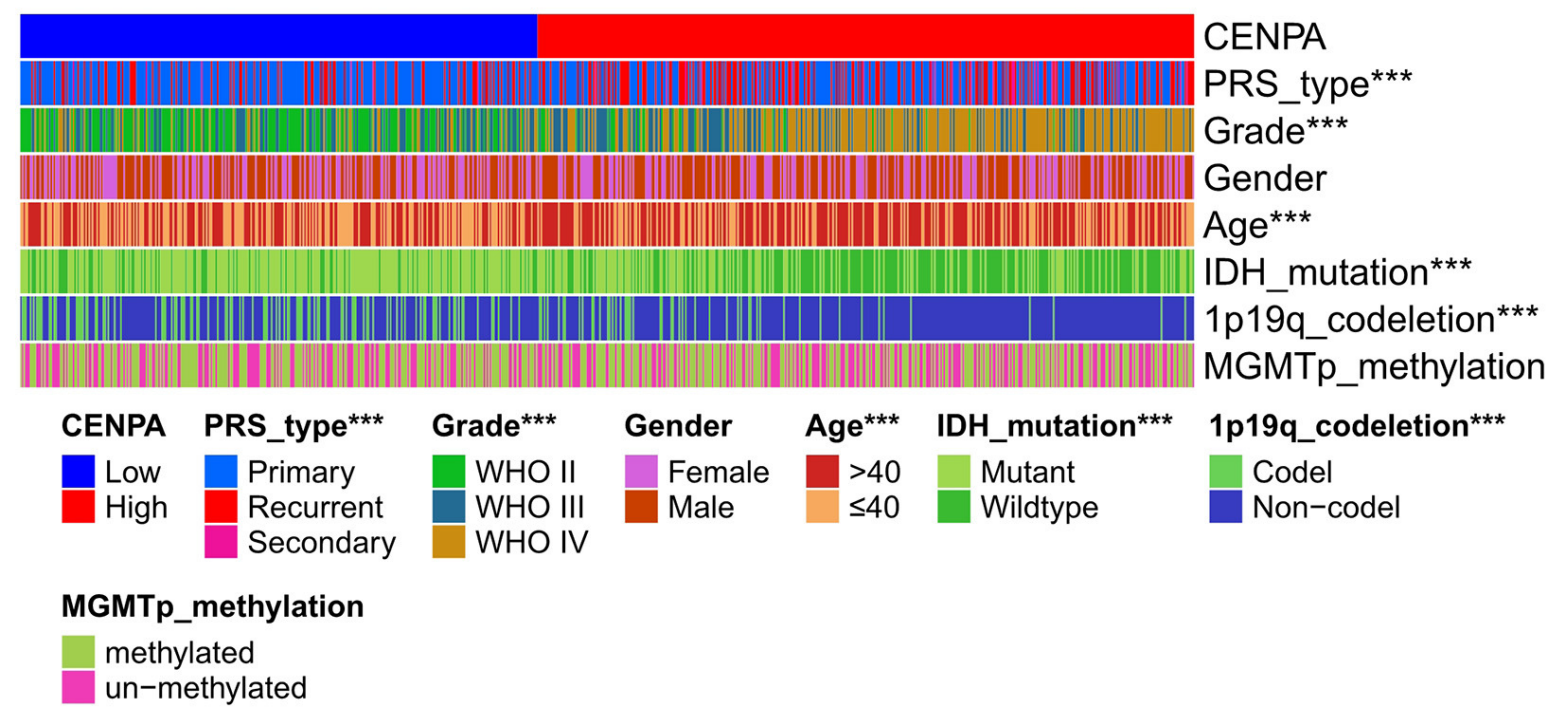
Heatmap of clinical pathological characteristics in high- and low-CENPA expression groups (chi-square test, *P < 0.05, **P < 0.01, ***P < 0.001).

A total of 687 samples with complete information extracted from the CGGA database (including LGG and GBM) were used for subsequent analysis. The result showed that the expression of CENPA was significantly correlated with the grade, age, PRS type, IDH mutation and 1p19q codeletion status (Figure 4).

### Overall survival curves for the expression of CENPA in different glioma subtypes

**Figure 5:**
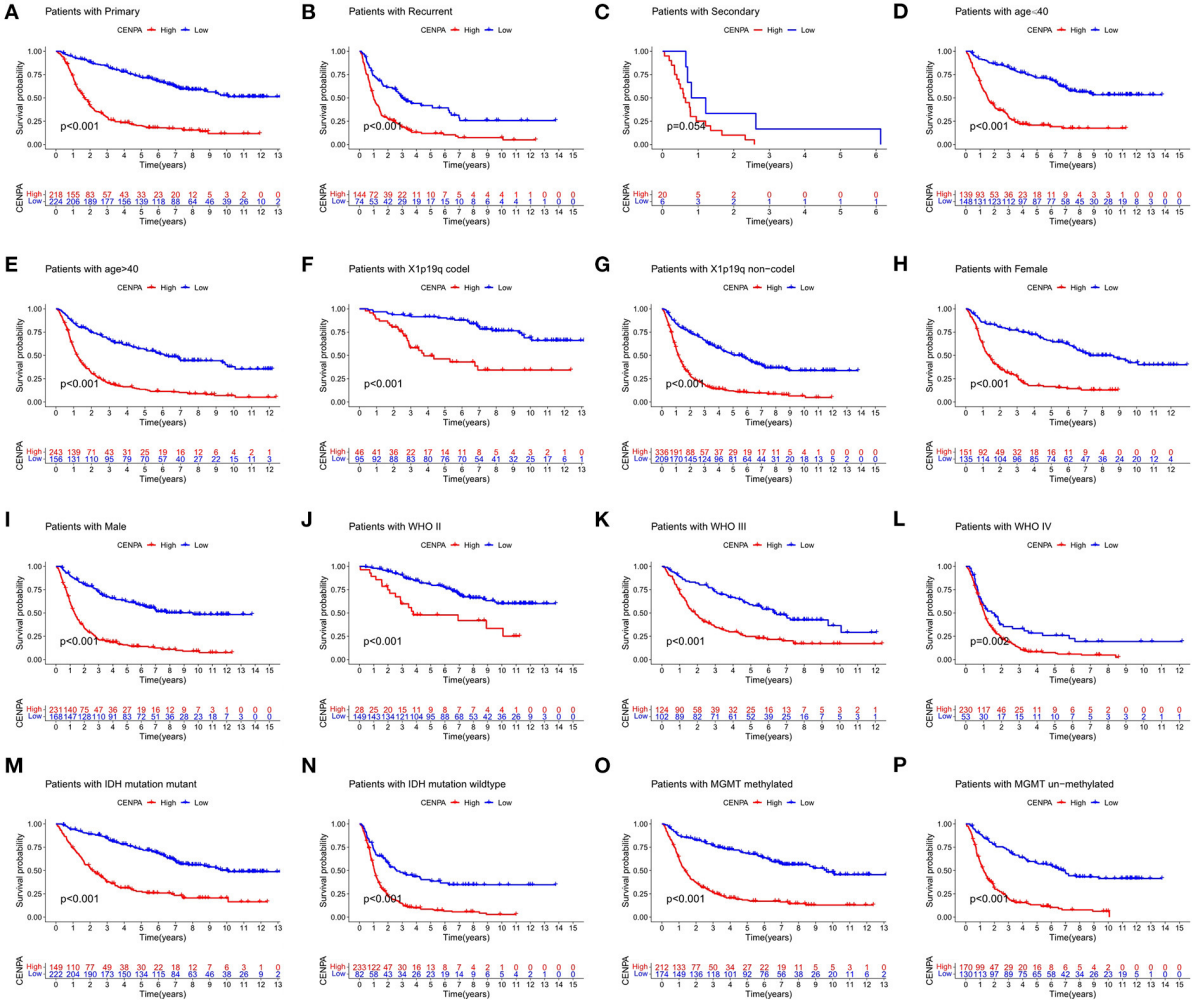
CENPA remains prognostic in different subgroups of patients with glioma, including **(A–C)** PRS type, **(D,E)** age, **(F,G)** 1p19q status, **(H,I)** sex, **(J–L)** WHO grade, **(M,N)** IDH status, and **(O,P)** MGMT status.

To further validate the prognostic value of CENPA in other subgroups, we conducted KM survival analysis in different subgroups. As is shown in Figure 5, high expression of CENPA significantly affected the survival of patients of different ages (Figures 5D,E), 1p19q status (Figures 5F,G), sex (Figures 5H,I), WHO grade (Figures 5G–L), IDH status (Figures 5M,N), and MGMT status (Figures 5O,P). In addition, high expression of CENPA suggested a poor prognosis in patients with primary (Figure 5A) and recurrent (Figure 5B) gliomas. The same trend was observed in patients with secondary glioma (Figure 5C). The results demonstrated that high expression of CENPA in different subgroups implied a poor prognosis for the patients.

### Enrichment analysis of CENPA

**Figure 6:**
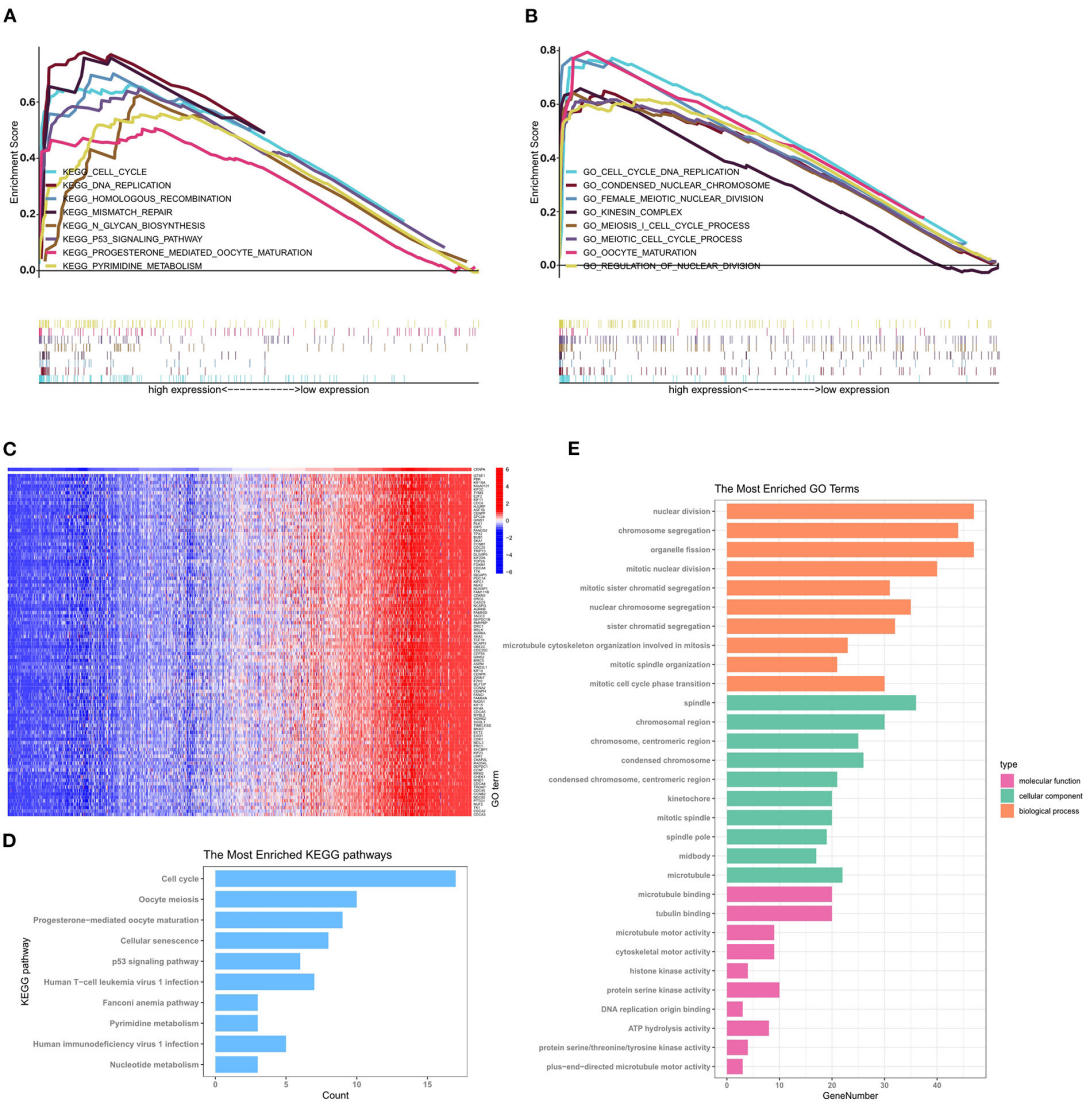
GSEA, GO analysis, and KEGG enrichment analysis reveal the potential mechanism by which CENPA promotes glioma progression using the CGGA database (LGG+GBM). The top 8 gene ontologies **(A)** and KEGG pathways **(B)** based on the normalized enrichment score (NES) between the high- and low-CENPA expression phenotypes. **(C)** Pearson correlation coefficient heatmap of the top 100 genes positively related to CENPA. The top 10 KEGG pathways **(D)** and biological processes, cellular components, and molecular functions **(E)** of the top 100 genes co-expressed with CENPA in gliomas.

GSEA was performed to further identify differentially expressed pathways and GO between the low- and high-CENPA expression groups. A total of 18 significant gene sets and biological processes were discovered using the cutoff criteria of |ES| > 0.5 and a *p* < 0.05 (Supplementary Table 1). The eight most significant enriched GO and signaling pathways were selected for visualization according to the normalized enrichment score (NES). As shown in Figures 6A,B, KEGG analysis revealed that CENPA was significantly enriched in “cell cycle,” “DNA replication,” “homologous recombination,” “mismatch repair,” “N-glycan biosynthesis,” “P53 signaling pathway,” “progesterone-mediated oocyte maturation,” and “pyrimidine metabolism.” The results of GO analysis were similar to those of KEGG analysis, where CENPA was significantly enriched in several mitosis-related functions. GO and KEGG enrichment analyses were performed to further understand the biological processes involved in CENPA in gliomas and the pathways it may affect. Using data from the CGGA database (LGG+GBM), we screened for genes co-expressed with CENPA with Pearson correlation coefficients >0.6 and *p* < 0.005. A total of 418 genes co-expressed with CENPA were finally screened out (Supplementary Table 2). A heatmap was constructed showing the top 100 genes co-expressed with CENPA based on the Pearson correlation coefficient in glioma (Figure 6C). KEGG analysis of top 100 genes co-expressed with CENPA enriched in the cell cycle, oocyte meiosis, progesterone-mediated oocyte maturation, cellular senescence, p53 signaling pathway, human T-cell leukemia virus 1 infection, Fanconi anemia pathway, pyrimidine metabolism, human immunodeficiency virus 1 infection, and nucleotide metabolism (Figure 6D). The annotations of the GO terms suggested the top 100 genes co-expressed with CENPA were involved in biological processes such as nuclear division, chromosome segregation, organelle fission, mitotic nuclear division, mitotic sister chromatid segregation, and nuclear division.

The results of GSEA, KEGG analysis, and GO enrichment analysis suggested that abnormal expression of CENPA in patients with glioma might affect the normal progression of mitosis and DNA repair, resulting in promoting glioma development.

### Relationship between CNEPA immune infiltration, temozolomide sensitivity, and CENPA

**Figure 7:**
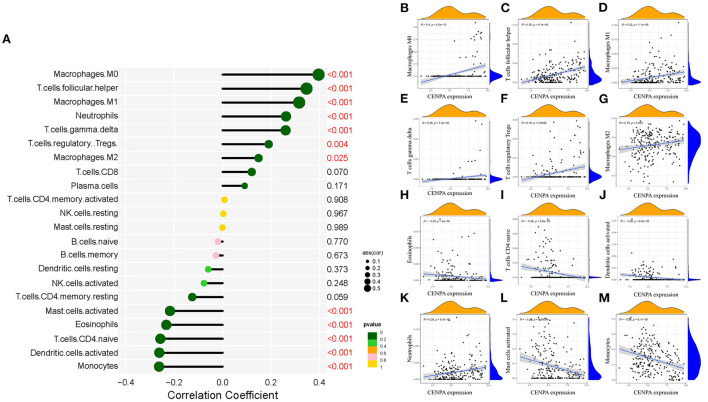
Correlation between CENPA expression and immune cell filtration in the CGGA database and TCGA database. **(A)** Correlation between CENPA expression and 22 kinds of immune infiltration cells in TCGA database. **(B–M)** Scatter plots of correlation between CENPA and significantly related immune-infiltrating cells (*p* < 0.05). Pearson coefficient was used for the correlation test.

**Figure 8:**
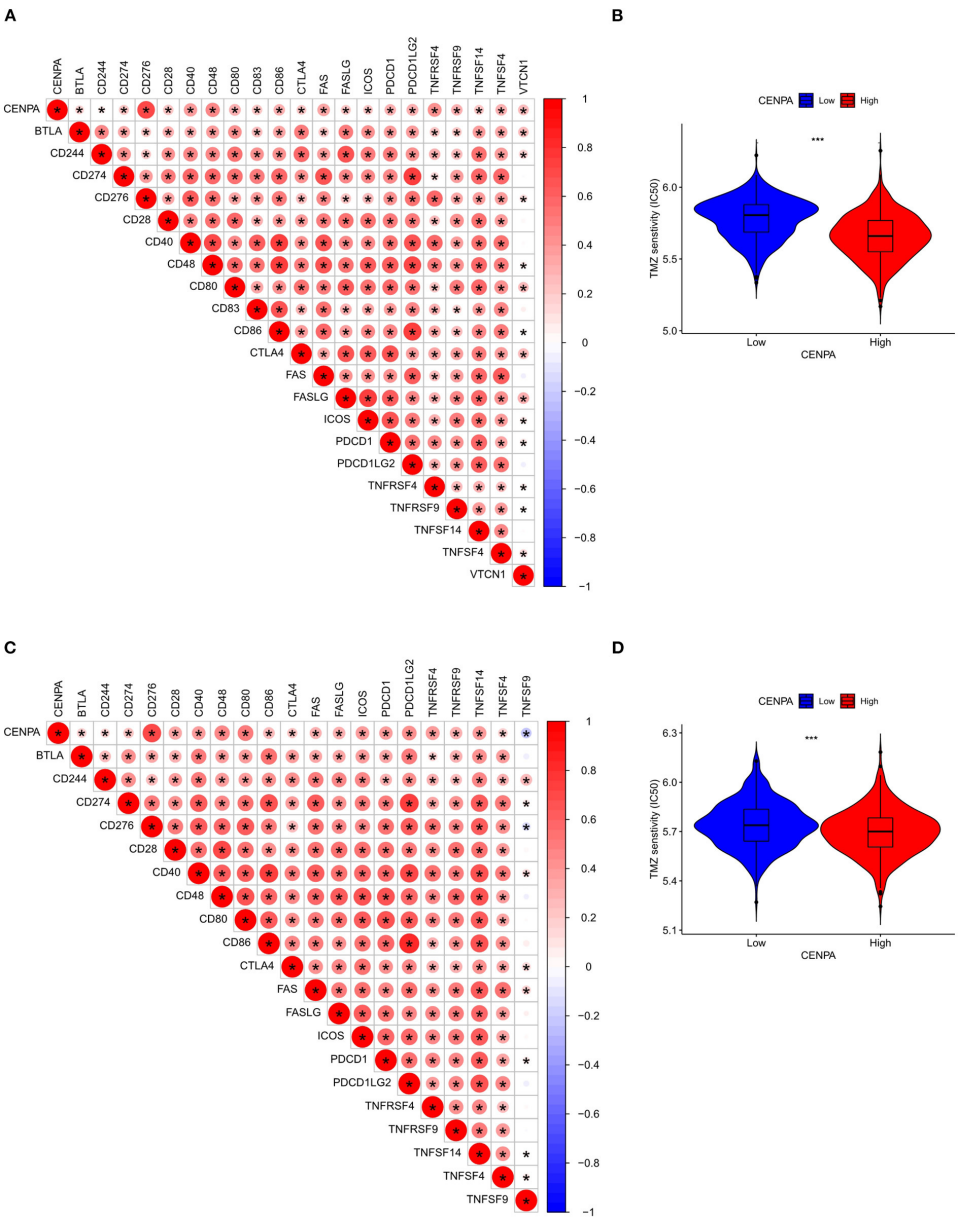
Assessment of the correlation between CENPA and immune features in gliomas. CENPA significantly correlated with immune checkpoints in the CGGA database **(A)** and TCGA database **(C)**. Sensitivity of temozolomide significantly differs between the low- and high-CENPA-expression group using the CGGA database **(B)** and TCGA database **(D)** (Wilcoxon test, *P < 0.05, **P < 0.01, ***P < 0.001). The Pearson coefficient was used for the correlation test.

Immune-infiltrating cells, as an important component of the tumor microenvironment, play a significant role in tumor behavior and disease prognosis (20). The CIBERSORT package was used to estimate the correlation between CENPA expression and diverse immune cells. As for glioma patients in the TCGA dataset, CENPA expression was found to be positively correlated with macrophage M0, T follicular helper cells, macrophage M1, neutrophils, gamma delta T cells, Tregs, macrophage M2, CD8 T cells, and plasma cells, and inversely related to monocytes, activated dendritic cells, CD4 naive T cells, eosinophils, activated mast cells, and resting memory CD4 T cells (Figure 7, Table 1). Further analysis using the ESTIMATE package showed that stromal score, immune score, and estimate score were significantly higher in the high-CENPA expression group than in the low-expression group in the data retrieved from both TCGA (Supplementary Figure 1A) and CGGA (Supplementary Figure 1B) databases. It was suggested that CENPA was highly implicated in immune infiltration and the formation of multiple components in gliomas. Finally, we explored the correlations between CENPA and immune checkpoints (21). The result showed that CENPA was positively correlated with BTLA, CD274, CD244, CD276, CD28, CD40, CD48, CD80, CD86, CTLA4, FAS, FASLG, ICOS, PDCD1, PDCD1LG2, TNFRSF4, TNFRSF9, TNFSF14, and TNFSF4 in the data retrieved from TCGA (Figure 8A) and CGGA (Figure 8C), which suggested a potential synergy of CENPA with known immune checkpoints. In general, these findings revealed that CENPA was associated with immune cell infiltration, immune score, and immune checkpoints in patients with glioma. The IMvigor210 cohort (22) was used to explore the underlying association between CENPA expression and cancer immunotherapy. Although short-term survival was higher in patients with low CENPA expression than in those with high expression, the opposite was true in patients with an overall survival beyond 5 years with borderline significance (*P* = 0.051) (Supplementary Figure 2A). Meanwhile, the response rate in patients with high CENPA expression was significantly higher than that in patients with low CENPA expression in the IMvigor 210 cohort (Supplementary Figure 2B). In summary, patients with high expression of CENPA had a better response to immunotherapy and had better long-term benefits for immunotherapy. We further evaluated the correlation between different CENPA expressions and patients' TMZ sensitivity in the data retrieved from the CGGA and TCGA databases using the pRRophetic package (23), respectively. The high-CENPA expression group was more sensitive to TMZ in the data retrieved from TCGA (Figure 8B) and CGGA (Figure 8D) databases. In summary, CENPA can be used as an indicator to guide patients undergoing chemotherapy and immunotherapy.

**Table 1:** Analysis of the correlation between CENPA and immune-infiltrating cells.

**Cell**	**cor**	***p*-value**
B cells naive	−0.01953	0.769759
B cells memory	−0.02815	0.673128
Plasma cells	0.091268	0.170572
T cells CD8	0.12043	0.070134
T cells CD4 naive	−0.2573	8.80E-05
T cells CD4 memory resting	−0.12557	0.058898
T cellsCD4 memory activated	0.007754	0.90751
T cells follicular helper	0.346911	8.08E-08
T cells regulatory Tregs.	0.190611	0.003946
T cells gamma delta	0.260391	7.18E-05
NK cells resting	0.002766	0.966948
NK cells activated	−0.07702	0.247786
Monocytes	−0.2628	6.12E-05
Macrophages M0	0.39688	5.53E-10
Macrophages M1	0.316513	1.13E-06
Macrophages M2	0.148623	0.025136
Dendritic cells resting	−0.05946	0.372522
Dendritic cells activated	−0.26177	6.55E-05
Mast cells resting	−0.00088	0.989455
Mast cells activated	−0.21735	0.00098
Eosinophils	−0.23282	0.000404
Neutrophils	0.262027	6.44E-05

Cor, Pearson correlation coefficient.

### Single-cell analysis of CENPA

**Figure 9:**
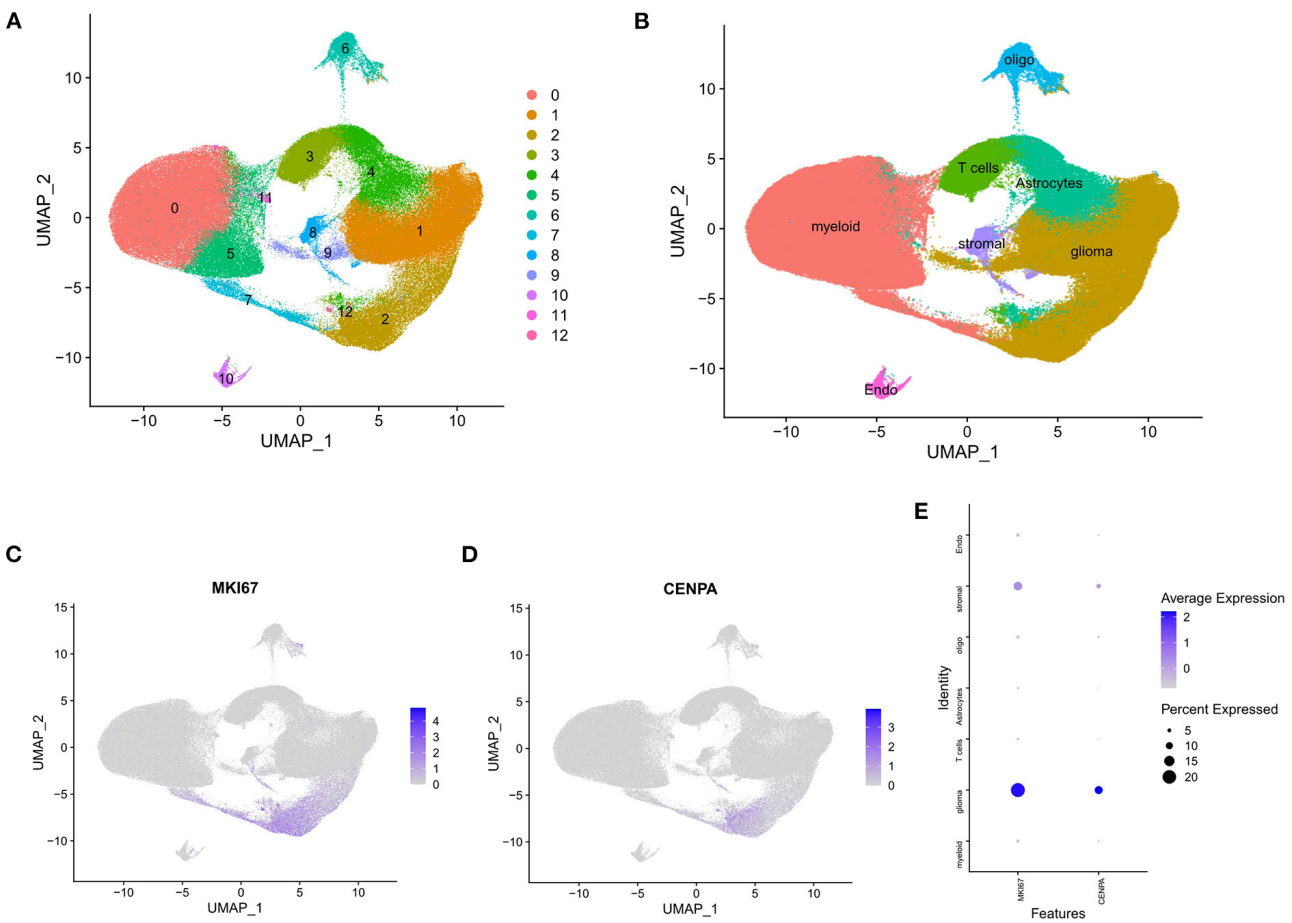
Single-cell analysis of single-cell mRNA sequence. **(A)** Two-dimensional UMP plot depicted that 250,944 single cells were divided into 13 clusters and displayed in different colors. **(B)** The 13 clusters were annotated as seven types of cells: glioma, oligodendrocytes, astrocytes, myeloid cells, T cells, stromal cells, and endothelial cells. **(C)** MKI67 expression in each cell. **(D)** CENPA expression in each cell. **(E)** Bubble plot showed the expression of MKI67 and CENPA in different types of cells.

The raw data were processed using the Seurat package, and all cells were divided into 13 clusters (Figure 9A) and finally annotated as seven types of cells: glioma, oligodendrocytes, astrocytes, myeloid cells, T cells, stromal cells, and endothelial cells (Figure 9B). Scatter plots and bubble plots showed that MKI67 and CENPA were mainly co-expressed in glioma and stromal cells (Figures 9C–E), which revealed that CENPA-expressing cells showed high proliferation. This is consistent with the results of the enrichment analysis and CCK8 experiment. Results of both enrichment and single-cell analyses indicated that mitosis was more intense in glioma cells from patients with glioma with high CENPA expression, which may explain the negative correlation between patient survival and CENPA expression.

### Cox analysis based on the expression of CENPA

**Figure 10:**
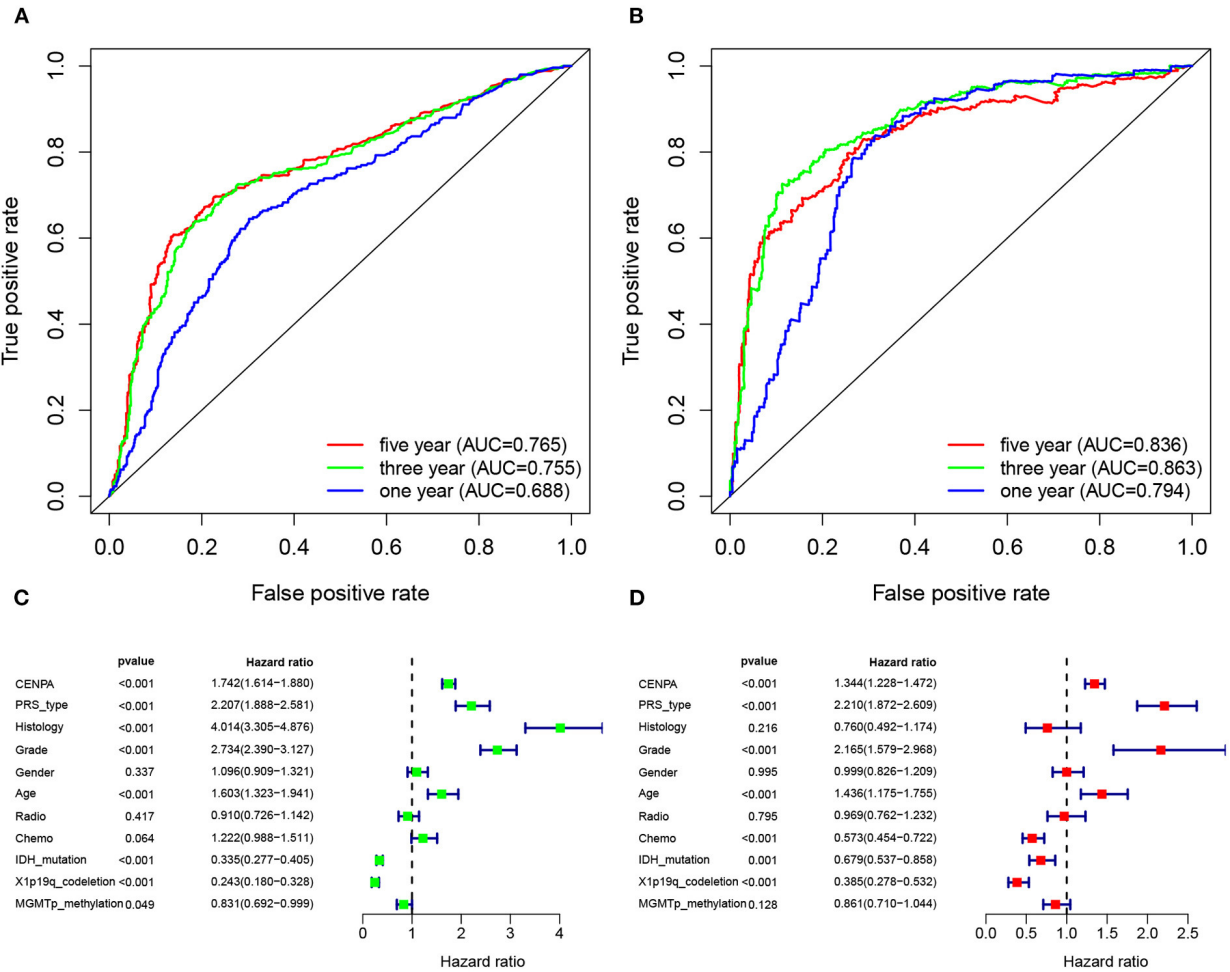
CENPA expression can be used to determine patient prognosis. The receiver operator characteristic curve analysis of CENPA uses the CGGA database **(A)** and TCGA database **(B)**. Univariate analysis **(C)** and multivariate analysis **(D)** of CENPA using the CGGA database.

The receiver operating characteristic curve analysis was performed using data from the CGGA and TCGA databases, respectively. The results of ROC analysis of CENPA using the CGGA database showed that CENPA was a predictor of 1-year (AUC = 0.688), 3-year (AUC = 0.755), and 5-year (AUC =0.765) survival (Figure 10A). The results using TCGA database also showed that CENPA was a predictor of 1-year (AUC = 0.794), 3-year (AUC = 0.863), and 5-year (AUC = 0.836) survival (Figure 10B). Univariate Cox analysis showed that CENPA (HR = 1.742; 95% CI = 1.614–1.880; *p* < 0.001), along with PRS type, histology, grade, and age, was a high-risk factor. By contrast, IDH mutation, 1p19q codeletion, and MGMT methylation were low-risk factors (Figure 10C). Multivariate Cox analysis revealed that CNEPA (HR = 1.344; 95% CI = 1.228–1.472; *p* < 0.001) was independently associated with overall survival, suggesting that CENPA could serve as an independent prognostic indicator for glioma. PRS type, grade, age, chemotherapy, IDH mutation, and 1p19q codeletion may also be independent prognostic markers (Figure 10D).

## Discussion

Gliomas exhibit a relatively poor prognosis among primary central nervous system tumors. Clinical characteristics in the traditional sense, such as age and tumor stage, are important factors affecting clinical outcomes (24). As research continues, molecular typing, such as mutations in IDH and codeletion of chromosome arms 1p and 19q (1p/19q codeletion), is also used as an important indicator of the prognosis in patients with glioma. The analysis of the data extracted from the CGGA database in the current study also confirms that the prognosis of glioma is associated with the aforementioned factors. However, due to the complex molecular regulation and cellular heterogeneity of gliomas, the clinical outcomes of patients with the same cancer stage may differ (25). Therefore, new methods are urgently needed to determine the prognosis of patients more accurately.

The CENP family is considered an important functional gene in tumorigenesis. Previous studies have shown that high expression of CENPH leads to poor prognosis in patients with cervical carcinoma. Similarly, high expression of CENPM promotes the development of breast cancer, whereas CENPF serves as an oncogene in breast cancer development (26–28). CENPA, as a mitophagy-specific variant, is considered a key epigenetic marker for mitophagy recognition and replication (29). On the one hand, it is necessary for the formation and maintenance of mitotic granules. On the other hand, it forms the platform for kinetochore assembly and mediates chromatin segregation (30, 31). Abnormal expression of CENPA may lead to chromosomal mismatches, disrupting genomic integrity and eventually promoting tumor development (32, 33). Previous studies have revealed that CENPA is highly expressed in multiple cancer tissues, such as prostate cancer, hepatocellular carcinoma, invasive breast cancer, and colorectal cancer (32, 34–36). CENPA expression was found to be upregulated in glioma in this study. Then, using data downloaded from the CGGA database, we performed a KM survival analysis to confirm CENPA prognostic efficacy in different glioma subgroups. Experiments *in vivo* revealed that high CENPA could promote glioma progression. Meanwhile, we also analyzed the relationship between CENPA and the clinical characteristics of patients with glioma. In addition, GSEA, KEGG analysis, GO analysis, and immune infiltration analysis were performed to explore the possible mechanisms between elevated CENPA expression and glioma progression. CENPA is primarily expressed in cells during the cell cycle, according to single-cell sequencing data. Finally, we performed a Cox analysis based on the expression of CENPA.

KM survival analysis showed that low CENPA expression was associated with a better prognosis in patients with glioma. The 5-year survival rate for the high-CENPA expression group was only 11.13% (54/485), but it was 44.94% (218/465) for the low-CENPA expression group. CENPA also showed good survival efficacy in different subgroups. We found a significant correlation between CENPA expression and age, grade, PRS type, IDH mutation, and 1p10q codeletion status. A strong correlation with numerous glioma prognostic risk factors suggests that CENPA may play an important role in glioma progression. The analysis of data retrieved from TCGA TARGET GTEx database, GSE4290, and GSE16011 revealed a significant difference in the expression of CENPA between normal and tumor tissues, and the higher the grade, the higher the expression of CENPA. Similarly, qPCR and IHC results of samples obtained from our institute also support the high expression of CENPA in glioma samples.

Combining the results of GSEA, GO analysis, and KEGG enrichment analysis, abnormal expression of CENPA may promote glioma progression by interfering with the normal process of mitosis. It has been demonstrated that MKI67 is related to cell proliferation and the active phases of the cell cycle (37). MKI67 is commonly used as a cell proliferation marker in the clinical management of glioma (38). Notably, single-cell analysis of CENPA showed that CENPA and MKI67 are expressed in the same type of cells, mainly in glioma cells, suggesting that the high expression of CENPA means that the cells are in an active proliferative state. This is consistent with the results of cellular experiments and enrichment analysis and may also explain why survival is worse in patients with high CENPA expression.

An additional important finding in this study is that the expression of CENPA correlated with the degree of immune infiltration in glioma. We found that CENPA expression was positively correlated with the degree of macrophage M0, follicular helper T cells, macrophage M1, neutrophils, gamma delta T cells, T cells, regulatory Tregs, and macrophage M2. Meanwhile, CENPA expression was negatively correlated with activated mast cells, eosinophils, CD4 naive T cells, activated dendritic cells, and monocytes. Dendritic cells (DCs) are the most powerful antigen-presenting cells, which play an important role in the induction of anti-tumor immunity (39, 40). The dendritic cell vaccine has been used in clinical trials (41). Treg cells can suppress anti-tumor immunity, thereby impeding protective immune surveillance of the tumor and hindering an effective anti-tumor immune response in the tumor host, thereby promoting cancer progression (42, 43). The negative correlation between CENPA and DCs and positive correlation between CENPA and Tregs indicate weaker anti-tumor immunity in patients with glioma with high CENPA expression. Immune checkpoint receptors and their cognate ligands are expressed on the surface of immune cells and tumor cells, respectively. Immune checkpoint expression is upregulated in tumors and contributes to the immune evasion of tumor cells (44). Herein, it was revealed that there was a significant positive correlation between CENPA and immune checkpoint expression. Notably, there was significant co-expression between CENPA and CD276 (Pearson correlation coefficients >0.7 both in TCGA and CGGA databases). CD276 has been documented to mediate glioma immune escape and increase glioma aggressiveness (45, 46). Immunotherapy targeting CD276 may be effective in patients with glioma with high expression of CENPA. Furthermore, the ESTIMATE results exposed significant differences in estimate score, immune score, and stromal score in the high- and low-CENPA expression groups in both the CGGA and TCGA databases. According to the results of IMgvior210, the survival rate of patients with high CENPA expression is higher among those who receive immunotherapy. Patients with high CENPA expression also have a higher response rate to immunotherapy than patients with low CENPA expression. This suggests that patients with high CENPA expression have better efficacy for immunotherapy. Drug sensitivity analysis suggests that patients with high CENPA expression are more sensitive to temozolomide. In summary, CENPA could be used as a reference for the clinical treatment of patients.

In addition, ROC curve analysis using the CGGA and TCGA databases both shows that the AUC values for CNEPA at 1, 3, and 5 years were almost >0.7, indicating CENPA as a predictor of survival. The results of univariate and multivariate Cox analyses showed that CENPA was a high-risk factor and could be used as an independent prognostic indicator in patients with glioma.

Finally, the ROC and Cox analyses showed that CENPA has good prognostic efficacy.

## Conclusion

Taken altogether, the results revealed that CENPA expression is upregulated in glioma and can be used as a prognostic marker and potential therapeutic target in patients with glioma.

## Data availability statement

The original contributions presented in the study are included in the article/Supplementary materials, further inquiries can be directed to the corresponding authors.

## Ethics statement

The studies involving human participants were reviewed and approved by the Ethics Committee at Zhongnan Hospital of Wuhan University. The patients/participants provided their written informed consent to participate in this study.

## Author contributions

XL and JC contributed to conception and design and study supervision. BW, SL, and LW performed the experiments and analyzed the data. MA, BY, and DW prepared the figures and tables. WW, ZheL, and ZhiL drafted the manuscript and revised it critically. All authors contributed to the article and approved the submitted version.

## Funding

This work was supported by the National Natural Science Foundation of China (NSFC 82171326) and the Research Fund from Medical Sci-Tech Innovation Platform of Zhongnan Hospital, Wuhan University (PTXM2021006).

## Conflict of interest

The authors declare that the research was conducted in the absence of any commercial or financial relationships that could be construed as a potential conflict of interest.

## Publisher's note

All claims expressed in this article are solely those of the authors and do not necessarily represent those of their affiliated organizations, or those of the publisher, the editors and the reviewers. Any product that may be evaluated in this article, or claim that may be made by its manufacturer, is not guaranteed or endorsed by the publisher

## Supplementary material

Supplementary Figure 1 ESTIMATE analysis of glioma patients with different CENPA expressions in TCGA **(A)** and CGGA **(B)** datasets.

Supplementary Figure 2 Validation of CENPA in the IMvigor210 cohort. **(A)** Kaplan–Meier analysis of patients with different CENPA expressions. **(B)** Comparison of response rates between high CENPA expression and low CENPA expression group.
