# Supplementary figures and images for: CENPA acts as a prognostic factor that relates to immune infiltrates in gliomas
Source: Front Neurol. 2022 Oct 19;13:1015221. doi: 10.3389/fneur.2022.1015221 (PMC9626989; doi:10.3389/fneur.2022.1015221)

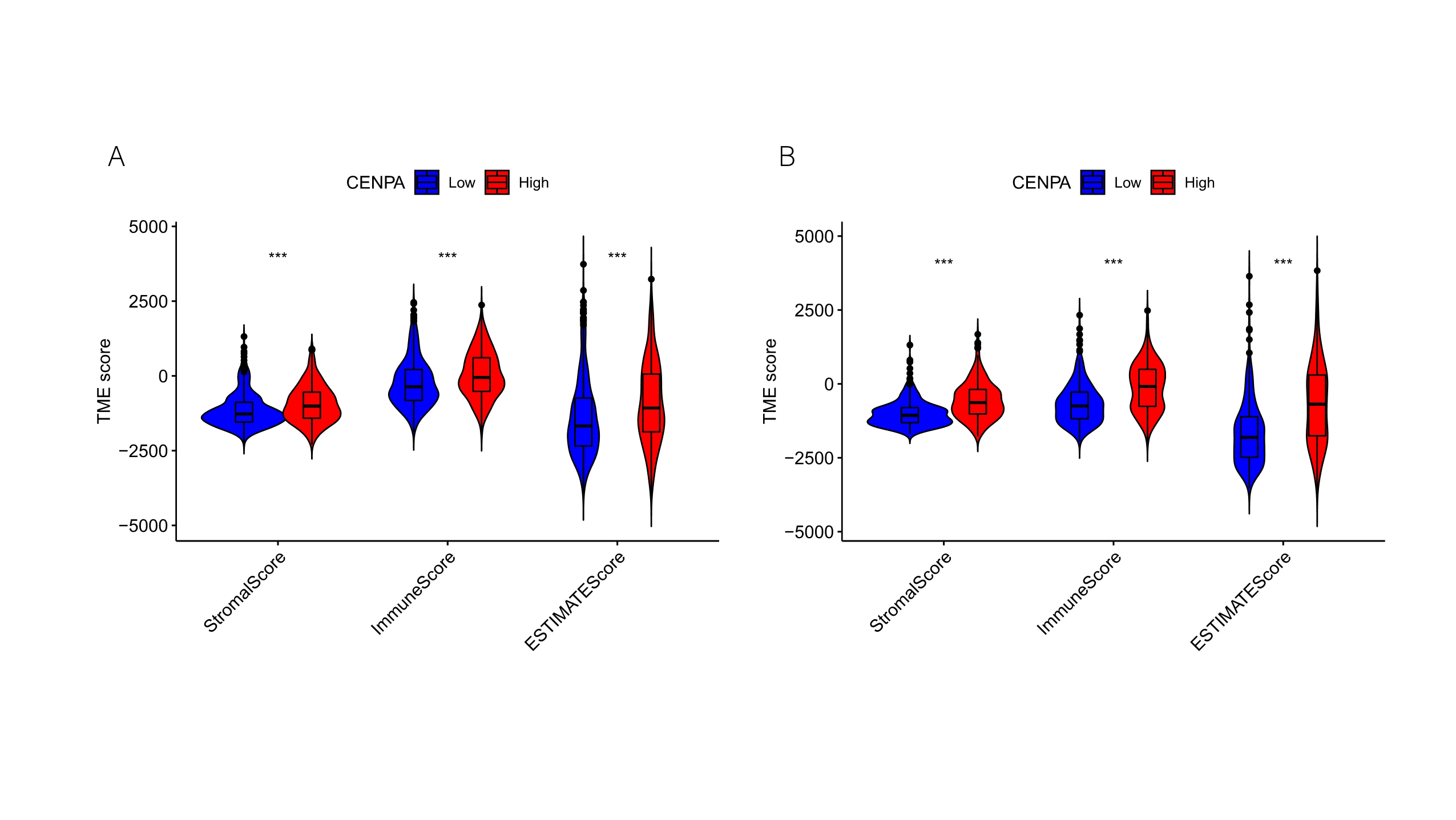

Supplement: Supplementary Figure 1 — ESTIMATE analysis of glioma patients with different CENPA expressions in TCGA (A) and CGGA (B) datasets. [file Image_1.JPEG]

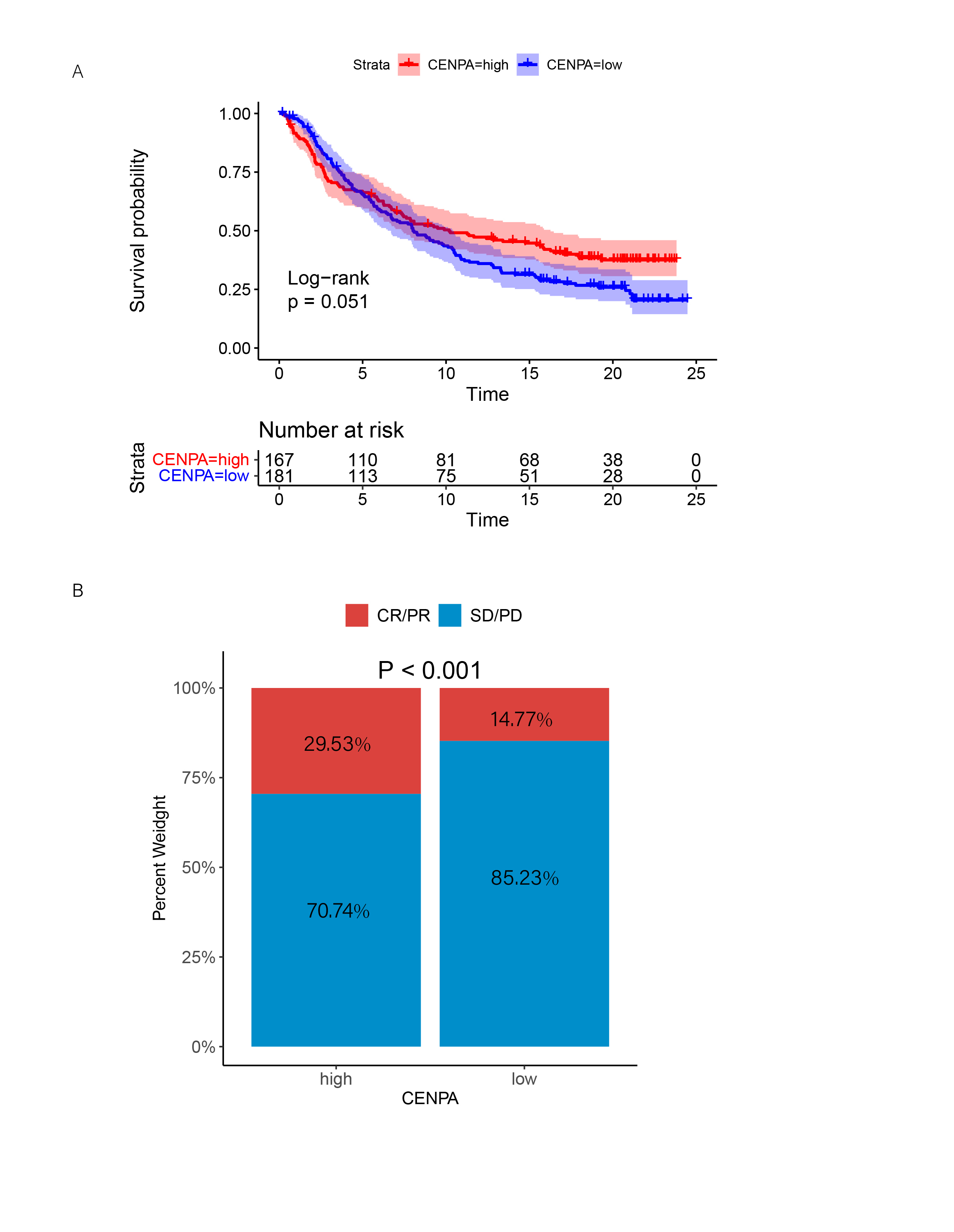

Supplement: Supplementary Figure 2 — Validation of CENPA in the IMvigor210 cohort. (A) Kaplan–Meier analysis of patients with different CENPA expressions. (B) Comparison of response rates between high CENPA expression and low CENPA expression group. [file Image_2.JPEG]
