# Supplementary material for: CENPA acts as a prognostic factor that relates to immune infiltrates in gliomas
Source: Front Neurol. 2022 Oct 19;13:1015221. doi: 10.3389/fneur.2022.1015221 (PMC9626989; doi:10.3389/fneur.2022.1015221)
Supplement: Supplementary file 3 [file Table_1.DOCX]

Table S1 The gene set enriches the high CENPA expression phenotype. Gene sets with NOM p value < 0.05 and | ES | ＞0.5 are considered as significant.

| NAME | ES | NES | NOM pval |
| --- | --- | --- | --- |
| DNA_REPLICATION | 0.777 | 1.88 | 0 |
| MISMATCH_REPAIR | 0.757 | 1.83 | 0 |
| HOMOLOGOUS_RECOMBINATION | 0.701 | 1.81 | 0.002 |
| KEGG_CELL_CYCLE | 0.662 | 1.93 | 0 |
| P53_SIGNALING_PATHWAY | 0.647 | 2.01 | 0 |
| N_GLYCAN_BIOSYNTHESIS | 0.623 | 1.81 | 0 |
| BASE_EXCISION_REPAIR | 0.612 | 1.69 | 0.002 |
| RNA_POLYMERASE | 0.603 | 1.62 | 0.018 |
| NUCLEOTIDE_EXCISION_REPAIR | 0.594 | 1.68 | 0.012 |
| SPLICEOSOME | 0.592 | 1.68 | 0.02 |
| AMINOACYL_TRNA_BIOSYNTHESIS | 0.591 | 1.57 | 0.023 |
| RNA_DEGRADATION | 0.582 | 1.69 | 0.008 |
| PYRIMIDINE_METABOLISM | 0.558 | 1.78 | 0.002 |
| RIG_I_LIKE_RECEPTOR_SIGNALING_PATHWAY | 0.548 | 1.66 | 0.012 |
| PANTOTHENATE_AND_COA_BIOSYNTHESIS | 0.545 | 1.54 | 0.036 |
| BLADDER_CANCER | 0.524 | 1.68 | 0.016 |
| SMALL_CELL_LUNG_CANCER | 0.514 | 1.65 | 0.014 |
| PROGESTERONE_MEDIATED_OOCYTE_MATURATION | 0.506 | 1.74 | 0.006 |

ES: enrichment score; NOM: nominal; NES: normalized enrichment score.
