# Supplementary material for: CENPA acts as a prognostic factor that relates to immune infiltrates in gliomas
Source: Front Neurol. 2022 Oct 19;13:1015221. doi: 10.3389/fneur.2022.1015221 (PMC9626989; doi:10.3389/fneur.2022.1015221)
Supplement: Supplementary file 4 [file Table_2.DOCX]

Table S2 Genes co-expression with CENPA with a threshold of Pearson correlation coefficient >0.6 and P value<0.005

| Gene | cor | pvalue |
| --- | --- | --- |
| ACTL6A | 0.617585839 | 4.35E-108 |
| ADAMTS7 | 0.631355713 | 2.56E-114 |
| AGBL5 | 0.614124793 | 1.43E-106 |
| AK2 | 0.609655714 | 1.23E-104 |
| ALYREF | 0.665182735 | 4.79E-131 |
| APEX2 | 0.614470595 | 1.01E-106 |
| ARHGAP11A | 0.770746325 | 4.12E-201 |
| ARHGAP11B | 0.705530075 | 4.04E-154 |
| ARHGEF39 | 0.784691026 | 3.11E-213 |
| ASF1B | 0.915667022 | 0 |
| ASPM | 0.911937356 | 0 |
| ATAD2 | 0.706678961 | 7.8E-155 |
| ATAD5 | 0.646923407 | 9.43E-122 |
| AUNIP | 0.688990024 | 3.34E-144 |
| AURKA | 0.875558938 | 2.96439387504748e-323 |
| AURKB | 0.912253977 | 0 |
| BIRC5 | 0.922248783 | 0 |
| BORA | 0.736039177 | 2.34E-174 |
| BRCA1 | 0.756742571 | 8.96E-190 |
| BRCA2 | 0.616562831 | 1.23E-107 |
| BRIP1 | 0.784160332 | 9.35E-213 |
| BTG3 | 0.647008468 | 8.56E-122 |
| BUB1 | 0.873409291 | 9.43072504781771e-320 |
| BUB1B | 0.81625227 | 2.36E-244 |
| BYSL | 0.614036274 | 1.57E-106 |
| C11orf82 | 0.816452896 | 1.43E-244 |
| C11orf84 | 0.662785291 | 8.66E-130 |
| C16orf59 | 0.674426734 | 5.23E-136 |
| C17orf53 | 0.776217675 | 9.2E-206 |
| C18orf54 | 0.689835269 | 1.08E-144 |
| C19orf40 | 0.726876425 | 5.44E-168 |
| C19orf48 | 0.640111065 | 1.91E-118 |
| C1orf112 | 0.621830143 | 5.65E-110 |
| C21orf58 | 0.660825352 | 9.07E-129 |
| C2orf44 | 0.602333496 | 1.54E-101 |
| C2orf48 | 0.618195859 | 2.34E-108 |
| C4orf46 | 0.661044355 | 6.98E-129 |
| C5orf34 | 0.755964871 | 3.63E-189 |
| C8orf76 | 0.615237299 | 4.69E-107 |
| CARHSP1 | 0.687987516 | 1.27E-143 |
| CASC5 | 0.831842242 | 4.89E-262 |
| CASP2 | 0.618970131 | 1.06E-108 |
| CASP3 | 0.609122974 | 2.07E-104 |
| CASP6 | 0.609868143 | 9.94E-105 |
| CCDC15 | 0.609292871 | 1.75E-104 |
| CCDC150 | 0.712620381 | 1.38E-158 |
| CCDC18 | 0.728351686 | 5.35E-169 |
| CCDC77 | 0.675204545 | 1.96E-136 |
| CCNA2 | 0.897865465 | 0 |
| CCNB1 | 0.880776978 | 0 |
| CCNB2 | 0.93648339 | 0 |
| CCNE1 | 0.623711505 | 8.05E-111 |
| CCNF | 0.823259364 | 4.37E-252 |
| CCNJL | 0.658035732 | 2.49E-127 |
| CD276 | 0.67335199 | 2.02E-135 |
| CDC20 | 0.897886464 | 0 |
| CDC25A | 0.806490183 | 4.07E-234 |
| CDC25B | 0.625095586 | 1.91E-111 |
| CDC25C | 0.918865171 | 0 |
| CDC45 | 0.870498684 | 4.44763416063948e-315 |
| CDC6 | 0.874552976 | 1.27468936627042e-321 |
| CDC7 | 0.709610325 | 1.13E-156 |
| CDCA2 | 0.897154154 | 0 |
| CDCA3 | 0.896297915 | 0 |
| CDCA4 | 0.847398592 | 1.35E-281 |
| CDCA5 | 0.877989788 | 0 |
| CDCA7 | 0.740874104 | 7.97E-178 |
| CDCA7L | 0.698747794 | 5.7E-150 |
| CDCA8 | 0.924871854 | 0 |
| CDK1 | 0.880575335 | 0 |
| CDK2 | 0.750267162 | 8.66E-185 |
| CDKN2C | 0.766192272 | 2.45E-197 |
| CDKN3 | 0.853972835 | 1.57E-290 |
| CDT1 | 0.760952548 | 4.23E-193 |
| CENPE | 0.791866011 | 7.89E-220 |
| CENPF | 0.886792452 | 0 |
| CENPH | 0.839635621 | 1.42E-271 |
| CENPI | 0.740137686 | 2.72E-177 |
| CENPK | 0.847544758 | 8.64E-282 |
| CENPL | 0.768471235 | 3.25E-199 |
| CENPM | 0.797144224 | 7.51E-225 |
| CENPN | 0.808683157 | 2.3E-236 |
| CENPO | 0.773866089 | 9.53E-204 |
| CENPW | 0.809007898 | 1.06E-236 |
| CEP55 | 0.882004908 | 0 |
| CEP89 | 0.698071474 | 1.46E-149 |
| CHAF1A | 0.799073792 | 1.01E-226 |
| CHAF1B | 0.768656485 | 2.28E-199 |
| CHEK1 | 0.835511142 | 1.83E-266 |
| CHEK2 | 0.767998809 | 7.99E-199 |
| CHRNA5 | 0.602589198 | 1.21E-101 |
| CKAP2 | 0.673998519 | 8.96E-136 |
| CKAP2L | 0.869238248 | 4.33478989617706e-313 |
| CKAP4 | 0.622457557 | 2.95E-110 |
| CKS2 | 0.795602859 | 2.28E-223 |
| CLP1 | 0.604511148 | 1.88E-102 |
| CLSPN | 0.740733393 | 1.01E-177 |
| CNIH4 | 0.678860477 | 1.88E-138 |
| COQ2 | 0.622623477 | 2.49E-110 |
| CRNDE | 0.653630662 | 4.32E-125 |
| CTB-175P5.4 | 0.662156966 | 1.84E-129 |
| CTD-2267D19.6 | 0.72012748 | 1.82E-163 |
| CTNNAL1 | 0.605904009 | 4.86E-103 |
| CTPS1 | 0.73108538 | 6.98E-171 |
| DAXX | 0.617079252 | 7.28E-108 |
| DBF4 | 0.782626785 | 2.21E-211 |
| DCLRE1B | 0.660897897 | 8.31E-129 |
| DDOST | 0.618508781 | 1.7E-108 |
| DDX11 | 0.64151154 | 4.05E-119 |
| DDX12P | 0.679506985 | 8.2E-139 |
| DDX20 | 0.617506498 | 4.72E-108 |
| DDX39A | 0.697731181 | 2.33E-149 |
| DEK | 0.623852098 | 6.96E-111 |
| DEPDC1 | 0.866169228 | 2.48E-308 |
| DEPDC1B | 0.860517061 | 7.15E-300 |
| DESI2 | 0.675435716 | 1.47E-136 |
| DIAPH3 | 0.635924923 | 1.87E-116 |
| DKC1 | 0.629114168 | 2.78E-113 |
| DLGAP5 | 0.885162534 | 0 |
| DNMT1 | 0.710804142 | 1.98E-157 |
| DNMT3B | 0.622475167 | 2.9E-110 |
| DONSON | 0.603197776 | 6.7E-102 |
| DOT1L | 0.602664013 | 1.12E-101 |
| DRAXIN | 0.64268899 | 1.1E-119 |
| DSCC1 | 0.672207502 | 8.43E-135 |
| DSN1 | 0.800507936 | 3.96E-228 |
| DTL | 0.800017521 | 1.2E-227 |
| DTYMK | 0.655602479 | 4.34E-126 |
| E2F1 | 0.818132671 | 2.14E-246 |
| E2F2 | 0.822101148 | 8.75E-251 |
| E2F7 | 0.72062201 | 8.59E-164 |
| E2F8 | 0.764482883 | 6.08E-196 |
| ECT2 | 0.836870784 | 3.92E-268 |
| EFNB1 | 0.671000953 | 3.79E-134 |
| ELAVL1 | 0.649839463 | 3.41E-123 |
| EME1 | 0.802631472 | 3.13E-230 |
| EPHB2 | 0.609118895 | 2.08E-104 |
| ERCC6L | 0.801705933 | 2.6E-229 |
| ESCO2 | 0.784188376 | 8.82E-213 |
| ESPL1 | 0.804229598 | 7.88E-232 |
| EXO1 | 0.871227586 | 3.07920781422194e-316 |
| EZH2 | 0.822975862 | 9.12E-252 |
| FAM111A | 0.603038438 | 7.82E-102 |
| FAM111B | 0.820830888 | 2.28E-249 |
| FAM136A | 0.650918014 | 9.91E-124 |
| FAM64A | 0.871963095 | 2.0466274126457e-317 |
| FAM72C | 0.639407703 | 4.14E-118 |
| FAM72D | 0.767960219 | 8.6E-199 |
| FAM83D | 0.82895118 | 1.27E-258 |
| FANCA | 0.670295709 | 9.08E-134 |
| FANCC | 0.808217102 | 6.94E-236 |
| FANCD2 | 0.819511916 | 6.57E-248 |
| FANCE | 0.609394597 | 1.59E-104 |
| FANCI | 0.822203027 | 6.73E-251 |
| FBXO43 | 0.605384839 | 8.06E-103 |
| FBXO5 | 0.767671796 | 1.49E-198 |
| FEN1 | 0.727883026 | 1.12E-168 |
| FKBP7 | 0.643966791 | 2.63E-120 |
| FOXD2-AS1 | 0.605923078 | 4.77E-103 |
| FOXM1 | 0.898762308 | 0 |
| GABPB1 | 0.611294803 | 2.42E-105 |
| GAS2L3 | 0.739752658 | 5.16E-177 |
| GEN1 | 0.712928729 | 8.78E-159 |
| GGH | 0.707467001 | 2.51E-155 |
| GINS1 | 0.845509711 | 4.17E-279 |
| GINS2 | 0.844911322 | 2.52E-278 |
| GINS4 | 0.617442701 | 5.04E-108 |
| GJC1 | 0.709308307 | 1.75E-156 |
| GLE1 | 0.613152199 | 3.8E-106 |
| GMPS | 0.683269478 | 6.31E-141 |
| GPX7 | 0.751667131 | 7.46E-186 |
| GSG2 | 0.751658854 | 7.57E-186 |
| GTSE1 | 0.87657482 | 0 |
| H2AFV | 0.615917334 | 2.36E-107 |
| H2AFX | 0.656369152 | 1.77E-126 |
| H2AFY | 0.605934667 | 4.72E-103 |
| H2AFZ | 0.646091374 | 2.41E-121 |
| HAUS1 | 0.729395422 | 1.03E-169 |
| HAUS5 | 0.626292521 | 5.45E-112 |
| HAUS8 | 0.736040014 | 2.34E-174 |
| HDGF | 0.622020818 | 4.64E-110 |
| HELLS | 0.781178278 | 4.27E-210 |
| HJURP | 0.905265701 | 0 |
| HMGB2 | 0.75938544 | 7.46E-192 |
| HMGB3 | 0.660319746 | 1.66E-128 |
| HMGN2 | 0.643647103 | 3.76E-120 |
| HMMR | 0.810169384 | 6.62E-238 |
| HN1L | 0.653812607 | 3.5E-125 |
| HNRNPAB | 0.701773478 | 8.31E-152 |
| HSPG2 | 0.600283756 | 1.1E-100 |
| IGFBP2 | 0.664678712 | 8.82E-131 |
| IKBIP | 0.664207232 | 1.56E-130 |
| ILF2 | 0.622167368 | 3.99E-110 |
| IQGAP3 | 0.818962551 | 2.64E-247 |
| IRX5 | 0.605197202 | 9.67E-103 |
| ISL2 | 0.603783051 | 3.81E-102 |
| ITGB3BP | 0.713402162 | 4.36E-159 |
| KATNA1 | 0.600318892 | 1.06E-100 |
| KDELC1 | 0.697619954 | 2.72E-149 |
| KDELR2 | 0.616498056 | 1.31E-107 |
| KDM1A | 0.611242852 | 2.55E-105 |
| KHDRBS1 | 0.616075792 | 2.01E-107 |
| KIAA0101 | 0.869483528 | 1.78470912609624e-313 |
| KIAA0922 | 0.627873864 | 1.03E-112 |
| KIF11 | 0.84615839 | 5.87E-280 |
| KIF14 | 0.853903557 | 1.96E-290 |
| KIF15 | 0.848839241 | 1.62E-283 |
| KIF18A | 0.827211353 | 1.34E-256 |
| KIF18B | 0.80368697 | 2.76E-231 |
| KIF20A | 0.855900522 | 3.11E-293 |
| KIF20B | 0.679411256 | 9.27E-139 |
| KIF23 | 0.860389394 | 1.1E-299 |
| KIF24 | 0.614912003 | 6.5E-107 |
| KIF2C | 0.931136644 | 0 |
| KIF4A | 0.902483288 | 0 |
| KIFC1 | 0.91720809 | 0 |
| KLHDC8A | 0.613882421 | 1.83E-106 |
| KNSTRN | 0.732722361 | 5.06E-172 |
| KNTC1 | 0.737177493 | 3.63E-175 |
| KPNA2 | 0.810284108 | 5.03E-238 |
| LEPRE1 | 0.643730654 | 3.42E-120 |
| LIG1 | 0.643569393 | 4.1E-120 |
| LIN9 | 0.653676464 | 4.09E-125 |
| LMNB1 | 0.789449505 | 1.4E-217 |
| LMNB2 | 0.792712277 | 1.26E-220 |
| LRR1 | 0.820811373 | 2.4E-249 |
| LRRC42 | 0.62385494 | 6.94E-111 |
| MAD2L1 | 0.833837984 | 1.97E-264 |
| MAD2L2 | 0.630324369 | 7.7E-114 |
| MASTL | 0.650596824 | 1.43E-123 |
| MCM10 | 0.768767181 | 1.85E-199 |
| MCM2 | 0.810919521 | 1.09E-238 |
| MCM3 | 0.789612771 | 9.91E-218 |
| MCM4 | 0.605223799 | 9.42E-103 |
| MCM5 | 0.753514088 | 2.87E-187 |
| MCM6 | 0.766855323 | 7E-198 |
| MCM7 | 0.656056082 | 2.55E-126 |
| MCM8 | 0.690011975 | 8.52E-145 |
| MELK | 0.906468291 | 0 |
| MGME1 | 0.670046207 | 1.24E-133 |
| MIR4435-1HG | 0.618509746 | 1.7E-108 |
| MIS18A | 0.704874101 | 1.03E-153 |
| MKI67 | 0.874148788 | 5.86949987259401e-321 |
| MLF1IP | 0.896229697 | 0 |
| MLX | 0.603561494 | 4.72E-102 |
| MMS22L | 0.604812387 | 1.4E-102 |
| MND1 | 0.870275404 | 1.0045238621494e-314 |
| MRGBP | 0.628399812 | 5.92E-113 |
| MSH6 | 0.645883204 | 3.05E-121 |
| MTFR2 | 0.801972856 | 1.41E-229 |
| MXD3 | 0.715717122 | 1.4E-160 |
| MYB | 0.601960731 | 2.21E-101 |
| MYBL2 | 0.913513382 | 0 |
| NAA38 | 0.628582577 | 4.88E-113 |
| NANP | 0.62697248 | 2.67E-112 |
| NASP | 0.633022597 | 4.3E-115 |
| NCAPD2 | 0.680335835 | 2.82E-139 |
| NCAPG | 0.91162383 | 0 |
| NCAPG2 | 0.757725137 | 1.52E-190 |
| NCAPH | 0.903521025 | 0 |
| NDC1 | 0.712926883 | 8.8E-159 |
| NDC80 | 0.917664611 | 0 |
| NEIL3 | 0.825597992 | 9.62E-255 |
| NEK2 | 0.882496879 | 0 |
| NKIRAS2 | 0.706015971 | 2.02E-154 |
| NOL10 | 0.646810966 | 1.07E-121 |
| NOP14 | 0.633497549 | 2.58E-115 |
| NR2C2AP | 0.665047133 | 5.64E-131 |
| NRAS | 0.608770687 | 2.93E-104 |
| NRBP1 | 0.600205453 | 1.19E-100 |
| NRM | 0.744637718 | 1.41E-180 |
| NT5DC2 | 0.699387506 | 2.34E-150 |
| NUF2 | 0.892067073 | 0 |
| NUP37 | 0.625287028 | 1.56E-111 |
| NUP62 | 0.641893012 | 2.65E-119 |
| NUP85 | 0.632873004 | 5.05E-115 |
| NUSAP1 | 0.854799307 | 1.1E-291 |
| NXT1 | 0.630973503 | 3.85E-114 |
| ODC1 | 0.636470287 | 1.03E-116 |
| ODF2 | 0.692396956 | 3.44E-146 |
| OIP5 | 0.87048943 | 4.60052417789149e-315 |
| ORC1 | 0.878291664 | 0 |
| ORC6 | 0.828765774 | 2.09E-258 |
| P4HB | 0.605478943 | 7.35E-103 |
| PARPBP | 0.825624569 | 8.97E-255 |
| PAXIP1 | 0.621311629 | 9.63E-110 |
| PBK | 0.89182606 | 0 |
| PCNA | 0.731866299 | 2E-171 |
| PDIA4 | 0.685830713 | 2.2E-142 |
| PDIA5 | 0.603718958 | 4.05E-102 |
| PHF19 | 0.6026381 | 1.15E-101 |
| PIF1 | 0.724962657 | 1.08E-166 |
| PKN3 | 0.695578046 | 4.52E-148 |
| PLK1 | 0.890669415 | 0 |
| PLK4 | 0.802925575 | 1.59E-230 |
| PMM2 | 0.61573522 | 2.84E-107 |
| POC1A | 0.85378901 | 2.83E-290 |
| POLA2 | 0.65439762 | 1.77E-125 |
| POLD3 | 0.723896817 | 5.63E-166 |
| POLE | 0.613628039 | 2.36E-106 |
| POLE2 | 0.769192694 | 8.18E-200 |
| POLQ | 0.778847217 | 4.8E-208 |
| POLR2D | 0.634402282 | 9.72E-116 |
| PPIH | 0.629557294 | 1.74E-113 |
| PPM1G | 0.634357884 | 1.02E-115 |
| PPP4C | 0.610974572 | 3.32E-105 |
| PRC1 | 0.877403612 | 0 |
| PRIM1 | 0.606738069 | 2.15E-103 |
| PRIM2 | 0.650311585 | 1.99E-123 |
| PRPF4 | 0.621801384 | 5.82E-110 |
| PRR11 | 0.700540735 | 4.68E-151 |
| PSMC3IP | 0.72004904 | 2.06E-163 |
| PTBP1 | 0.707380892 | 2.84E-155 |
| PTK7 | 0.642346618 | 1.6E-119 |
| PTTG1 | 0.871457215 | 1.32321520943426e-316 |
| RACGAP1 | 0.759207877 | 1.03E-191 |
| RAD18 | 0.68014029 | 3.63E-139 |
| RAD51 | 0.895301177 | 0 |
| RAD51AP1 | 0.799829178 | 1.84E-227 |
| RAD54B | 0.672995242 | 3.15E-135 |
| RAD54L | 0.820411616 | 6.66E-249 |
| RBBP8 | 0.711891335 | 4.04E-158 |
| RBM15B | 0.610296273 | 6.51E-105 |
| RBM8A | 0.612691582 | 6.02E-106 |
| RCC1 | 0.768127572 | 6.25E-199 |
| RCC2 | 0.700644683 | 4.05E-151 |
| REEP4 | 0.610052049 | 8.29E-105 |
| RFC2 | 0.717296336 | 1.32E-161 |
| RFC3 | 0.694405397 | 2.24E-147 |
| RFC4 | 0.772865897 | 6.74E-203 |
| RFC5 | 0.605072786 | 1.09E-102 |
| RFWD3 | 0.623070064 | 1.57E-110 |
| RMI2 | 0.769498368 | 4.55E-200 |
| RNASEH2A | 0.770021879 | 1.67E-200 |
| RNF122 | 0.667473891 | 2.93E-132 |
| RP1-74M1.3 | 0.611545858 | 1.88E-105 |
| RP11-108L7.15 | 0.618834026 | 1.22E-108 |
| RP11-303E16.2 | 0.712453759 | 1.77E-158 |
| RP11-443B20.1 | 0.72648035 | 1.01E-167 |
| RP11-932O9.10 | 0.648127452 | 2.41E-122 |
| RP5-991G20.4 | 0.60647888 | 2.77E-103 |
| RPIA | 0.67169042 | 1.61E-134 |
| RPN2 | 0.601700477 | 2.83E-101 |
| RRM1 | 0.626803534 | 3.18E-112 |
| RRM2 | 0.856656144 | 2.64E-294 |
| RTKN2 | 0.722717847 | 3.47E-165 |
| SEC61A1 | 0.617312287 | 5.75E-108 |
| SERPINH1 | 0.664679665 | 8.81E-131 |
| SF3A3 | 0.633975989 | 1.54E-115 |
| SGOL1 | 0.891925044 | 0 |
| SGOL2 | 0.801614572 | 3.2E-229 |
| SHCBP1 | 0.851408462 | 5.4E-287 |
| SKA1 | 0.921107749 | 0 |
| SKA3 | 0.830159484 | 4.83E-260 |
| SKP2 | 0.648383249 | 1.8E-122 |
| SMARCE1 | 0.610263568 | 6.72E-105 |
| SMC4 | 0.759244593 | 9.64E-192 |
| SNRNP40 | 0.618446656 | 1.81E-108 |
| SNRPB | 0.687120045 | 4.01E-143 |
| SOX11 | 0.608249226 | 4.9E-104 |
| SPAG5 | 0.74454408 | 1.65E-180 |
| SPC24 | 0.820086529 | 1.53E-248 |
| SPC25 | 0.693081548 | 1.36E-146 |
| STIL | 0.732886833 | 3.89E-172 |
| STK38 | 0.640341133 | 1.48E-118 |
| TACC3 | 0.824604522 | 1.31E-253 |
| TARBP2 | 0.606688419 | 2.26E-103 |
| TCEB3 | 0.603878027 | 3.47E-102 |
| TCF19 | 0.832422237 | 9.93E-263 |
| TCF3 | 0.643318892 | 5.42E-120 |
| TEAD2 | 0.625563689 | 1.17E-111 |
| TGFB1I1 | 0.637369277 | 3.87E-117 |
| TGIF1 | 0.624879854 | 2.39E-111 |
| TICRR | 0.731201001 | 5.8E-171 |
| TIMELESS | 0.830607863 | 1.43E-260 |
| TK1 | 0.874206111 | 4.72326757424232e-321 |
| TMEM106C | 0.631513042 | 2.17E-114 |
| TMEM194A | 0.6764242 | 4.19E-137 |
| TMEM39B | 0.619031849 | 9.98E-109 |
| TMPO | 0.629049614 | 2.98E-113 |
| TMSB15A | 0.639355223 | 4.39E-118 |
| TOE1 | 0.648338502 | 1.89E-122 |
| TONSL | 0.734470684 | 3.01E-173 |
| TOP2A | 0.881975358 | 0 |
| TPX2 | 0.926590924 | 0 |
| TRA2B | 0.621726621 | 6.28E-110 |
| TRAIP | 0.718696519 | 1.6E-162 |
| TRIM24 | 0.62238334 | 3.19E-110 |
| TRIP13 | 0.86756931 | 1.73280695654356e-310 |
| TROAP | 0.898090605 | 0 |
| TTK | 0.862959665 | 1.76E-303 |
| TUBB | 0.714848849 | 5.11E-160 |
| TUBG1 | 0.646757001 | 1.14E-121 |
| TXLNA | 0.637843989 | 2.3E-117 |
| TYMS | 0.829400972 | 3.77E-259 |
| UBE2C | 0.916498881 | 0 |
| UBE2I | 0.617499059 | 4.75E-108 |
| UBE2S | 0.694916105 | 1.12E-147 |
| UBE2T | 0.80451889 | 4.03E-232 |
| UBTD2 | 0.612404978 | 8.01E-106 |
| USP1 | 0.604585663 | 1.75E-102 |
| VRK1 | 0.604688554 | 1.58E-102 |
| WDHD1 | 0.777092532 | 1.61E-206 |
| WDR62 | 0.855784153 | 4.53E-293 |
| WDR76 | 0.795111584 | 6.71E-223 |
| WDR77 | 0.613156813 | 3.78E-106 |
| WEE1 | 0.733881379 | 7.81E-173 |
| XRCC2 | 0.712638229 | 1.35E-158 |
| YBX1 | 0.616955111 | 8.26E-108 |
| YTHDF2 | 0.626081256 | 6.8E-112 |
| ZC3HC1 | 0.611374932 | 2.23E-105 |
| ZNF367 | 0.80300925 | 1.31E-230 |
| ZNF829 | 0.607662907 | 8.71E-104 |
| ZNF850 | 0.605260191 | 9.09E-103 |
| ZNF90 | 0.719492131 | 4.79E-163 |
| ZWILCH | 0.764103672 | 1.23E-195 |
| ZWINT | 0.860525905 | 6.94E-300 |

Cor: Pearson correlation coefficient
