# Supplementary material for: CENPA acts as a prognostic factor that relates to immune infiltrates in gliomas
Source: Front Neurol. 2022 Oct 19;13:1015221. doi: 10.3389/fneur.2022.1015221 (PMC9626989; doi:10.3389/fneur.2022.1015221)
Supplement: Supplementary file 5 [file Table_3.DOCX]

https://www.jianguoyun.com/p/DTPfuHQQ65jsChj0q9IEIAA
